# The reflexive imagery task: An experimental paradigm for neuroimaging

**DOI:** 10.3934/Neuroscience.2018.2.97

**Published:** 2018-03-07

**Authors:** Hyein Cho, Wei Dou, Zaviera Reyes, Mark W. Geisler, Ezequiel Morsella

**Affiliations:** 1 Department of Psychology, San Francisco State University, CA, USA; 2 Department of Neurology, University of California, San Francisco, CA, USA

**Keywords:** consciousness, cognitive control, involuntary processing, reflexive imagery task, stimulus control

## Abstract

High-level cognitions can be triggered into consciousness through the presentation of external stimuli and the activation of certain action sets. These activations arise in a manner that is involuntary, systematic and nontrivial. For example, in the Reflexive Imagery Task (RIT), subjects are presented with visual objects and instructed to not think of the names of the objects. Involuntary subvocalizations arise on roughly 80% of the trials. We review the findings from this paradigm, discuss neural findings that are relevant to the RIT, and present new data that further corroborate the reliability and robustness of the RIT, a paradigm that could be coupled with neuroimaging technologies. We developed an RIT variant in which two, non-focal objects are presented simultaneously. In previous RITs, visual objects were presented only one at a time, in the center of the screen, and subjects were instructed to focus on the center of the screen, where these objects were presented. Replicating the RIT effect, involuntary subvocalizations still occurred on a high proportion of trials (*M* = 0.78). An RIT effect arose for both objects on a considerable proportion of the trials (*M* = 0.35). These findings were replicated in a second experiment having a different sample of subjects. Our findings are relevant to many subfields of neuroscience (e.g., the study of high-level mental processes, attention, imagery and action control).

## 1. Introduction

Understanding the mechanisms underlying “entry into consciousness” (“entry”, for short [1],[2]) remains one of the most challenging puzzles in science [3].

Entry is influenced by various processes, including those that are voluntary (e.g., choosing to think about certain things) or attention-based (see review in Most et al. [4]). Recent research has begun to illuminate the nature of the various kinds of mechanisms underlying entry that is involuntary. This form of entry can arise from the salience, motion, novelty or incentive/emotional quality of the stimulus [9]. Involuntary entry can be of percepts, urges [10] or even high-level cognitions. Regarding high-level cognitions, their involuntary entry can arise as a consequence of the activation of *action sets*, the topic of the present project. An action set would be “when perceiving *X*, then do *Y*” [11]; for example, “when I see a mailbox, I must deposit the letter that I am carrying”. Regarding action sets, Ach [11] speaks of the example in which, after activating the action set to “add things” and being presented with the numbers five and three, there is the involuntary entry of the *conscious content*^1^ “eight”. In this way, entry of high-level conscious contents can arise from the activation of action sets (“set-based entry”, for short). Theorists (e.g., Freud [12]; Helmholtz [13]; James [14]; N. E. Miller [15]; Wegner [16]) have proposed that, during such entry, one is conscious of the product (e.g., the phonological form “eight”) of sophisticated and unintentional processes, but not of the processes themselves, a view that has recurred in the history of psychology (e.g., [17],[18]).^2^

These conclusions suggest that, in a neuroimaging study, if the experimenter controls the activation of set and the stimulus conditions, then entry could be controlled externally and predictably, in ways that are not trivial and that involve high-level contents. Such a study on entry could employ the Reflexive Imagery Task (RIT [23]), which we review, along with the relevant neural findings, in the next section. We conclude our review with the presentation of new data which further corroborate the reliability and robustness of the paradigm.

### 1.1. Reflexive Imagery Task

The RIT (see review in Bhangal et al. [24]) is based on a rich research tradition, stemming from the experimental approaches of Ach [11], Stroop [25], Wegner [16], and Gollwitzer [26]. The paradigm was developed to investigate experimentally the involuntary entry of high-level conscious contents. In the initial, most basic version of the task [23], subjects are instructed to not subvocalize (i.e., say in their head but not aloud) the names of objects (e.g., line drawings from Snodgrass & Vanderwart [27]). In Allen et al. [23], subjects were presented before each trial with the instruction, “Don't Think of the Name of the Object” before an object was presented for 4 s, during which time subjects indicated by button press if they happened to subvocalize the name of the object. On the majority of the trials (86% in Allen et al. [23]; 87% in Cho et al. [28]; 73% in Merrick et al. [29]), subjects fail to suppress such subvocalizations. To illustrate the basic version of the RIT effect, momentarily, we will present to you, the reader, an object enclosed within parentheses. Your task is to *not* subvocalize (i.e., “say in one's head”) the name of the object. Here is the stimulus (▴). When presented with these instructions (which induce a certain action set) and then presented with this stimulus, most people cannot suppress the conscious experience of the phonological form of the word “triangle”.

It is important to appreciate that this RIT effect requires the process of object naming, a sophisticated, multi-stage process in which only one of tens of thousands of phonological representations is selected for production in response to a stimulus (e.g., CAT yields /k/, /œ/, /t/; Levelt [30]). After the presentation of the stimulus, the RIT effect arises after a few moments (*M* = 1,451.27 ms [*SD* = 611.42] in Allen et al. [23]; *M* = 2,323.91 ms [*SD* = 1,183.01] in Cho et al. [28]; *M* = 1,745.97 ms [*SD* = 620.86] in Merrick et al. [29]). There are more complex versions of the task. For example, in one study, RIT effects arose even though the involuntary effect involved a word-manipulation task similar to the childhood game of pig Latin (e.g., “CAR” becomes “AR-CAY”). In this variant of the RIT [31], subjects were instructed to not transform stimulus words according to the rule. Nevertheless, involuntary transformations still arose on more than 40% of the trials. This set-based effect is noteworthy because the involuntary transformation of the word stimulus requires symbol manipulation, a complex operation which is known to be associated with frontal cortex [32].^3^

### 1.2. Validity of subjects' self-reports

The evidence suggests that the RIT effect is both robust and reliable. However, some important questions remain concerning the validity of the effect. For instance, one major criticism is that the paradigm relies on the technique of self-report. Self-reports can be inaccurate as a result of (a) inaccurate memories of fleeting conscious contents that lead to incorrect self-reports [33]; (b) subjects basing their reports on a strategy of how to comport oneself during an experiment (see discussion in Morsella et al. [34]). Evidence from neuroimaging studies suggests that subjects are not confabulating about the occurrence of these mental events. In these studies, subjects must report about the occurrence of involuntary conscious contents [35]–[39]. Strong behavioral evidence for subjects' self-reports stems from one variant of the RIT. In this variant [40], subjects indicated by button press the basic RIT effect but, in addition, they had to press another button if the involuntary subvocalization rhymed with a word held in mind. Accurate performance (> 80% mean accuracy across trials) on this rhyming task provided evidence that subjects experience involuntary subvocalizations of the name of the object, for detecting a rhyme requires the retrieval of either the whole object name or, at minimum, the coda of the object name.

### 1.3. Evidence that the effect resembles a reflex

Empirical evidence and theory, including Wegner's [41] model of *ironic processing*^4^ (see discussion of relationship between Wegner's [41] model and the RIT in Bhangal et al. [24]) suggest that, for subjects, the effect “just happens”. The effect does not seem to be an artifact of high-level strategic processes. Supporting this view, in one version of the RIT, subjects reported on the majority of trials that the involuntary subvocalizations felt “immediate” [42]. Separate evidence supports the notion that the effect is not an artifact of strategic processes. First, on many trials, the effect arises too quickly to be caused by strategic processing [23],[28]. Second, the RIT effect still arises under conditions of cognitive load, in which it is difficult for subjects to implement strategic processing [28]. Third, the effect habituates (i.e., is less likely to arise) after repeated presentation of the same stimulus object, which suggests that the RIT effect is activated in a reflex-like manner [43]. Last, the nature of the subvocalizations is influenced systematically by stimulus dimensions such as word frequency [42]. Such an artifact of experimental demand would require for subjects to know the ways in which word frequency should influence latencies in an object-naming experiment.

It is important to note that the RIT is “reflex-like”, but is not a true reflex. A true reflex possesses a magnitude that reflects the intensity of the stimulus, as in the case of the startle reflex. The RIT does not have this property. Instead, the RIT depends on the activation of high-level, involuntary sets.

### 1.4. The importance of set in set-based entry

It is important to note that, in the basic version of the RIT, it is unlikely that subjects would experience the phonological representations of the names of the objects that are perceived visually without the activation of the action set. The activation of the action set is somehow initiated by the instruction to not think of the name of the object. With this in mind, it is important to point out that, in Allen et al. [23], there was an *Incidental Naming* condition in which subjects were not provided with the “do not think” instruction that leads to ironic effects [41]. Instead, the condition involved no explicit instructions regarding naming or not naming. For this condition, involuntary subvocalization of the object names still arose on 99% of the trials (range = 80% to 100%). The effect was comparable even on the very first trial (31 [97%] out of 32 first trials). The Incidental Naming condition served to evaluate subjects' spontaneous subvocalization rates in response to the stimuli when having no obvious action set toward the stimuli. Of course, simply mentioning to subjects the possibility of naming will increase the likelihood of subvocalization, which is a limitation of this condition.^5^

### 1.5. Neural correlates of the RIT effect

Investigations on the neural correlates of cognitive control, phonological processing and involuntary cognitions (including ironic processing; see Footnote 4), suggest that, in the basic RIT effect, there might be the recruitment of at least three distinct neural mechanisms: Those associated with (*1*) the action set to not subvocalize the name of the object; (*2*) the detection of a discrepancy between desired performance and the involuntary effect; (*3*) the phonological representation of the object name.

Regarding 1, neuroimaging studies suggest that the action set to perform a simple action (such as to *not* subvocalize an object name or to follow another simple rule of behavior) involves prefrontal cortex [32],[47],[48] (see evidence from neurophysiological studies involving monkeys in 47). For example, in ironic processing, the effortful, *operating* process (see Footnote 4) involves the dorsolateral prefrontal cortex [37],[49]. This was observed in a study involving functional magnetic resonance (fMRI) imaging [37].

The RIT effect involves the detection of undesired conscious content, resulting, in part, from the activation of set and stimulus conditions (for an electroencephalography study on thought suppression, see [50]). Studies employing fMRI have revealed that such detection has been associated with the activities of the anterior cingulate cortex [37],[39],[49], a region that has been associated with cognitive control [51], including cognitive conflict [52], the detection of error-prone processing [an fMRI study, 53] and more inclusively, any form of inefficient processing [54] (the region is located on the medial surface of the frontal lobe and interconnected with many motor areas). Inefficient processing includes both error-prone and conflict-related processes (see [55]–[57] for discussions of the role of the anterior cingulate cortex, lateral prefrontal cortex and hippocampus in the suppression, not of involuntary subvocalizations, but of undesired memory retrieval. These studies [55]–[57] are based on data from fMRI).

Regarding 3, controversy continues to surround the identification of the neural correlates of the phonological representations that are activated by heard, spoken speech (e.g., [58],[59]) (see relevant data from transcranial magnetic stimulation in 59). Thus, at this stage of understanding, strong claims cannot be made regarding the neural correlates of subvocalized speech (see discussion in Buchsbaum [60]; Buchsbaum & D'Esposito [61]). Nevertheless, investigations in neuropsychology and neuroimaging (e.g., fMRI; 62) suggest that the neural correlates of phonological representations involve the left superior temporal cortex (including the superior temporal gyrus and sulcus) and a medley of other regions (supramarginal gyrus, inferior frontal gyrus, precentral gyrus [62]–[65]).

Buchsbaum [60] concludes that the subvocalization of speech is often associated with activations in both (a) motor-related regions in frontal cortex, such as the inferior frontal gyrus (for phonological planning) and the precentral gyrus (for motor programming); (b) perception-related regions that are associated with speech perception (e.g., superior temporal sulcus). Accordingly, Scott [66] presents evidence that, during the act of subvocalization, corollary discharge provides the conscious sensory content of one's inner speech [67]. In an electroencephalography study by Ford et al. [67], mismatches involving one's intended speech and what one actually hears oneself utter aloud are associated with decreased functional synchrony (a kind of communication) between frontal and temporal lobes.

It should be pointed out that it remains controversial whether the subvocalization of speech requires the activation of motor-related regions or whether subvocalized speech and other forms of auditory imagery can arise without these activations [58],[59],[61],[68]. Thus, today there is no conclusive evidence that, for example, lesions to motor areas associated with speech production eradicate the capacity for subvocalizing or other kinds of verbal imagery [69]–[72]. For some evidence of a necessary, causal role of motor areas in speech perception, see Schomers, Kirilina, Weigand, Bajbouj and Pulvermüller [59].

In summary, it is clear that much is known about the neural correlates of many of the component processes underlying the RIT effect. Hence, the RIT is a rich and fecund experimental paradigm for hypothesis-testing research studies in the field of neuroscience.

### 1.6. Replication and extension of the RIT

For this review of the RIT, we took the opportunity to complement previous findings with new data that further corroborate the reliability and robustness of the paradigm. In previous RITs, involuntary entry arose from the processing of one single, focal stimulus, one that was in the center of the subjects' visual field and was the focus of visual attention. No RIT variant to date has presented an array of stimuli and had the subject not focus on one of the objects. There is always the possibility that subjects, when presented with such a complex stimulus scene, in which more than one visual object is presented, may not experience any RIT effect or, at the least, may be much less likely to experience the effect on a given trial. This leads to the question, would an RIT effect still arise if (a) more than one stimulus object is presented simultaneously; (b) the stimuli in the task are not as focal as those of previous studies? Can the RIT effect survive in a multi-stimulus scenario? Would the effect arise on a large proportion (> 0.70) of the trials, as was found in previous studies (e.g., 0.86 in Allen et al. [23]; 0.87 in Cho et al. [28]; and 0.73 in Merrick et al. [29])? If so, then this would corroborate that the RIT effect is both a robust and reliable phenomenon, one capable of arising in stimulus scenes that resemble everyday scenarios more than those of previous RITs.

**Figure 1.**
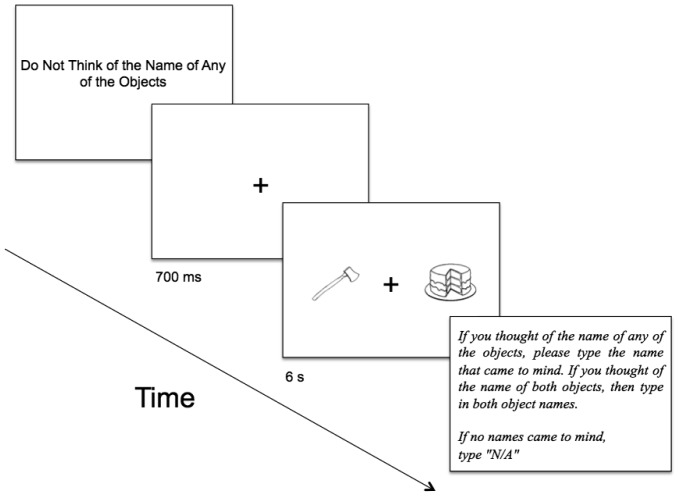
Schematic depiction of a trial (not drawn to scale).

To begin to investigate these questions, we developed a variant of the RIT in which, on each trial, two stimuli (visual objects) were presented (6 s) as a pair, with one stimulus being presented on the left side of the computer screen and one stimulus being presented on the right side of the screen (Figure 1). On each trial, subjects were instructed to focus on the fixation cross presented on the center of the screen and to not think of the names of any of the objects. Subjects indicated by button press if they happened to think of the name of any of the objects. Subjects pressed one button if they thought of the name of the object on the left, and they pressed another button if they thought of the name of the object on the right. If, during the duration of the trial, subjects thought of the name of any of the objects more than once, then they pressed the corresponding button each time that they experienced the thought. Unlike in previous studies, we examined the occurrence and latencies of all button presses. With this variant of the RIT, we took the opportunity to examine (a) whether the RIT effect still arises under so complex a circumstance, which is more complicated than that of previous studies; (b) whether subjects, on a given trial, experienced more than one involuntary subvocalization; (c) on a trial-by-trial basis, the latencies of the first subvocalization and rates of occurrence of all subsequent subvocalizations; (d) whether, because of the nature of reading (which is left to right), the spatial location (i.e., left versus right) of the object influenced the nature of our dependent measures.^6^

If more than one thought is triggered in this experimental context, then this is quite noteworthy, because it would be one of the first demonstrations of entry of more than one thought arising from external control. In addition, finding an RIT effect with our variant would corroborate the view that the RIT effect in other paradigms is not solely an artifact of subjects focusing on the critical stimulus. Moreover, if the RIT effect arises for each of the two objects presented on a given trial, then this would suggest that it is not the case that subjects' responding to one object hinders the ability of a response to the other object. This could occur if the involuntary subvocalization on a trial depletes the cognitive resources that are necessary for the involuntary subvocalization of the name of another object, at least during the 6 s span. To date, no RIT has taxed to this extent the processes involved in involuntary subvocalization.

Of import, our research project is the kind of incremental, *cumulative, theory-driven* research that leaders in the field of experimental psychology have recently encouraged [74],[75]. Moreover, the phenomenon at hand (the RIT effect and ironic processing) is a robust, multifaceted and reliable phenomenon that has been investigated for years, yielding the kind of programmatic research that is incremental and important for progress in the fields of psychological science and neuroscience [75]. In addition, the paradigm is perfectly suited for scanner-based neuroimaging research, because the task involves a simple procedure for presenting stimuli (e.g., a black-and-white line drawing), and because the dependent measure (the occurrence of involuntary mental imagery) does not require complicated movements on the part of the subject. Last, our task also provides a way of examining the mechanisms underlying entry into consciousness, one of the greatest enigmas in science [3],[76],[77]. The phenomenon of involuntary entry is of interest to many subfields within neuroscience, including consciousness, attention, self-regulation, psychopathology, mental imagery and mind wandering.

## 2. Method

### 2.1. Subjects

San Francisco State University students (*n* = 45; 37 females; *M_Age_* = 23.90 years; *SD_Age_* = 7.17 years) participated for course credit. The involvement of human subjects in our project was approved by the Institutional Review Board at San Francisco State University.

### 2.2. Stimuli and apparatus

Stimuli were presented on an Apple iMac computer monitor (50.8 cm) with a viewing distance of approximately 48 cm. Stimulus presentation and data recording were controlled by PsyScope software [78]. Subjects inputted their responses to questions and instructions by computer keyboard. All questions and instructions were written in black 36-point Helvetica font; all fonts and images were displayed in black hue on a light gray background. The stimuli consisted of 76 visual objects (Figure 1). Most of the stimuli were from Snodgrass and Vanderwart [27], while some were designed to resemble these Snodgrass images. These images were used successfully in previous research [23],[73],[79] (see Supplementary Table 1 for a list of the names of all of these objects). On each trial, two visual objects were presented concurrently in a side-by-side fashion with a fixation cross in between the visual objects (Figure 1). The array of stimuli, which was composed of both visual objects, was presented on the screen with a subtended visual angle of 17.76° × 5.96° (15 cm × 5 cm). Each object occupied the visual angle of 6.56°× 5.96° (5.5 cm × 5 cm).

### 2.3. Procedures

All subjects completed 38 trials in this modified version of the RIT. Each image was shown only once. For each trial, whether a given object appeared on the left side of the screen or on the right side of the screen was random. At the beginning of the experimental session, subjects were instructed that, on each trial, they would be shown two objects, with one object appearing on the left, and the other object appearing on the right. Subjects were instructed to not think of the name of any of the objects that were presented. If a subject did happen to think of the name of any of the objects, then the subject was instructed to indicate by button press each time that they happened to think of the name of any of the objects. It was emphasized to subjects that they should respond in this way as quickly as possible (trials in which RTs for a button press were less than or equal to 200 ms were excluded from analysis. This resulted in the loss of the data from only one trial). The presentation duration of the visual objects (6 s) was based on that of Allen et al. [23], with a longer duration allocated for the presentation of two objects (i.e., 50% more time was given from the original 4 second duration). If subjects did not happen to think of the name of any of the objects, then they did not respond in any way. Subjects were told that they could indicate by pressing the “z” key on the keyboard if they happened to think of the name of the object presented on the left of the fixation cross, and the character key “/” on the keyboard if they happened to think of the name of the object presented on the right of the fixation cross. In addition, subjects were informed that the keys correspond to the location of the object presentation (e.g., “z” key which is located on the left side of the keyboard for the objects presented on the left; “/” key which is located on the right side of the keyboard for the objects presented on the right). Both keys were covered with colored paper so that the keys could be easily distinguished from the other, neighboring keys. The “z” and “/” keys were chosen because (a) they are on opposite sides of a standard keyboard, thereby minimizing subjects' confusion; (b) the location of the keys are equidistant in relation to the spacebar. The pairing of keys to either spatial location on the screen were not counterbalanced because this could lead to undesired effects such as the Simon Effect [80].

Once subjects completed the experiment, they completed a set of psychological assessments^7^, as well as a series of funneled debriefing questions (following the procedures of Bargh & Chartrand [83]), which included general questions to assess whether subjects (a) were aware of the purpose of the study; (b) had any strategies for completing the task; (c) had anything interfere with their performance on the task; (d) knew the names of all of the presented objects; (e) thought of object names in a language other than English; (f) pressed the buttons in response to thinking of the names in another language. Additionally, subjects were asked questions regarding their performance on the task, to assess whether they (g) often thought of the names of both of the objects when the name of one object came to mind. These questions were included only to assess if a subject's data were acceptable for data analysis. From 52 subjects, data from 45 subjects were included in the analysis. The data for 7 subjects were excluded from analysis because (a) subjects did not follow instructions (e.g., looking away from the screen when stimuli were presented); (b) equipment malfunction (e.g., unexpected quitting of the computer software); (c) subjects did not press the button when they thought of the name of the object in a language other than English.

## 3. Results

Although the stimulus environment was more complicated than that of previous RITs, an RIT effect still emerged. The RIT effect is quantified as the proportion of trials on which an involuntary subvocalization arises in response to the presentations of the stimuli. The proportion of trials on which subjects had at least one involuntary subvocalization was 0.78 (*SD* = 0.20; *SE* = 0.03), a proportion that was significantly different from zero, *t* (44) = 26.39, *p* < 0.0001. This proportion is comparable to the proportions found in previous studies, which involved the presentation of only one stimulus at a time (e.g., 86% in Allen et al. [23]; 87% in Cho et al. [28]; 73% in Merrick et al. [29]). The same significant result was found with arcsine transformations of the proportion data, *t* (44) = 27.44, *p* < 0.0001. Arcsine transformations are often used to statistically normalize data that are in the form of proportions. Of the 45 subjects, 16 had an RIT effect on over 90% of the trials; 10 had an RIT effect on 80% to 90% of the trials; 9 had an RIT effect on 60% to 79% of the trials, and the percentages for the remaining 10 subjects were 58%, 58%, 58%, 53%, 53%, 50%, 45%, 37%, 37%, 34%. For trials on which there was an RIT effect, the mean latency of this effect was 2,493.57 ms (*SD* = 694.58, *SE* = 103.54). The latencies were comparable to those of previous studies (e.g., *M* = 1,451.27 ms [*SD* = 611.42] in Allen et al. [23]; *M* = 2,323.91 ms [*SD* = 1,183.01] in Cho et al. [28]; *M* = 1,745.97 ms [*SD* = 620.86] in Merrick et al. [29]).

The RIT effect occurred for both objects on a proportion of 0.35 of the trials (*SD* = 0.31; *SE* = 0.05), which was significantly different from zero, *t* (44) = 7.43, *p* < 0.0001, and was comparable to what was found in Merrick et al. [29], the only other RIT study in which two thoughts were triggered into the conscious field by external stimuli. This finding regarding an effect for both objects is also found with arcsine transformations of the proportion data, *t* (44) = 9.35, *p* < 0.0001.

If subjects experienced multiple stimulus-elicited thoughts during a trial, they then self-reported this by pressing the appropriate button each time that they had such a thought. The mean number of RIT effects per 6 s trial (that is, the RIT rate per trial) was 2.13 (*SD* = 3.03; *SE* = 0.45), with a range of 0.37 to 19.66. Whether an object appeared on the left of the screen or on the right of the screen did not affect this rate, *t* (44) = 0.07, *p* = 0.95, nor the likelihood of there being any RIT effect, *t* (44) = 1.12, *p* = 0.27.

### 3.1. Replication

We replicated our primary findings in a different sample from the same population (San Francisco State University students, *n* = 47). The procedures in this experiment were identical to those of the previous experiment, except that the number of stimuli was 72 instead of 76 and that the size of the stimuli was a bit larger: The array of stimuli, which was composed of both visual objects, was presented on the screen with a subtended visual angle of 34.32° × 11.75° (21 cm × 7 cm). Each object occupied the visual angle of 16.37° (9.78 cm × 9.78 cm). The proportion of trials on which subjects had at least one involuntary subvocalization was 0.91 (*SD* = 0.16; *SE* = 0.02), a proportion that was significantly different from zero, *t* (46) = 38.56, *p* < 0.0001. The RIT effect occurred for both objects on a proportion of 0.63 (*SD* = 0.27; *SE* = 0.04), which was significantly different from zero, *t* (46) = 15.61, *p* < 0.0001. If subjects experienced multiple RIT effects during a trial, they then self-reported this by pressing the appropriate button each time that they had such a thought. The mean number of RIT effects per 6 s trial (that is, the RIT rate per trial) was 4.67 (*SD* = 4.22; *SE* = 0.62), with a range of 0.36 to 19.63.

## 4. Discussion

Can an RIT with more than one (non-focal) stimulus elicit a sequence of involuntary, high-level thoughts? The present data suggest that the answer to this question is yes. An RIT effect (involuntary subvocalization) occurred for both objects on 35% of the trials, which is comparable to what was found in Merrick et al. [29], the only other RIT study in which two thoughts were triggered by external stimuli. The data are noteworthy because this is one of the first demonstrations of more than one thought being triggered through external control. The mean number of RIT effects per 6 s trial was 2.13, with a range of 0.37 to 19.66. These findings were replicated in an experiment having a different sample of subjects. Together, the data suggest that the mechanisms giving rise to involuntary subvocalization can be employed more than once within a short span.

The present experiment is the first RIT study in which more than one stimulus was presented simultaneously to the subject and in which the subject was not directly focusing visually on any of the critical stimuli. The RIT effect survived in such a (relatively) more complicated context, in which the perceptual scene contained more than one object and in which the subject was instructed to not look directly at any of the objects, which is unlike what occurred in previous RITs. On 78% of the 38 trials, subjects had at least one involuntary subvocalization. This percentage is comparable to the proportions found in previous studies, which involved the presentation of only one stimulus at a time (e.g., 86% in Allen et al. [23]; 87% in Cho et al. [28]; and 73% in Merrick et al. [29]). Moreover, the mean latency (∼2.5 s) of the first RIT effect per trial was comparable to that found in previous studies (e.g., *M* = 1,451.27 ms [*SD* = 611.42] in Allen et al. [23]; *M* = 2,323.91 ms [*SD* = 1,183.01] in Cho et al. [28]; *M* = 1,745.97 ms [*SD* = 620.86] in Merrick et al. [29]). The data also revealed that the likelihood of an effect seemed not to be influenced by whether an object was presented on the left of the screen or on the right of the screen. In short, our replication of both the latency data and the data regarding the likelihood of an RIT effect provides additional evidence that, even in contexts more complex than those of previous studies, the RIT is a robust and reliable phenomenon.

In line with our theoretical views, N. E. Miller [15] proposes that conscious content is more constrained than appears to be the case and that, under the right conditions, content activation can resemble reflexive, stimulus-to-response mappings. As noted above, elicitations of conscious content can be easier to control and predict than overt action [22]. From this point of view, the unpredictability of conscious contents in everyday life reflects, not the lack of external control, but rather the vagaries of quotidian stimulus scenes. As observed in our experiment, even multiple thoughts can be controlled externally in ways that are systematic and nontrivial. Regarding such constraint, it has been posited that conscious contents should be construed as highly constrained outputs of the nervous system [84]. These outputs are the result of “multiple constraint satisfaction” [5]. In our paradigm, the activation of conscious contents depended on a combination of both set and stimulus conditions. Regarding that which enters consciousness, these two factors could be deemed to be *determinant*, at least in our experimental arrangement. In this way, the RIT effect in our study builds on the important research by Gollwitzer [26] on “implementation intentions” (in which sets lead to automatic, stimulus-triggered behavioral dispositions) by demonstrating that, once certain sets are induced, responses to environmental stimuli can resemble reflex-like processes, even when the responses depend on sophisticated, unconscious mechanisms. Future investigations using the present variant of the RIT may examine the neural correlates of the various events involved during each trial (e.g., induction and maintenance of the task-related action sets and the entry into consciousness of the involuntary subvocalization).

### 4.1. The nature of the RIT effect

The RIT effect is a rich phenomenon that can be mined experimentally in many ways. We will not pretend to understand all aspects of what occurs in this effect, an effect that involves the involuntary entry into consciousness of high-level contents (see discussion in Allen et al. [23]). At the present stage of understanding, one can conclude the following. First, it seems that, for the involuntary effect to arise, the relevant action set must be activated. This activation stems from the instructions provided by the experimenter [23]. Without such an activation of set, it is unlikely that subjects would experience the phonological representations of the names of the objects that happen to be perceived visually. From at least the beginning of the trial until the onset of the visual object, the action set is then held in memory. During this time, the action set held in mind can be regarded as a case of *imageless thought*. This is because the action set influences behavioral dispositions without being maintained explicitly in consciousness [85] (see recent, relevant research in Scullin et al. [86]) (imageless thought was investigated first by theorists of the Würzburg School of Psychology [87]). During the trial, the final phenomenon of interest occurs when the appearance of a visual object begins the stages of processing that, somehow, leads to the consciousness of the involuntary imagery (e.g., subvocalization of the object name).

### 4.2. Implications for theory

The RIT effect corroborates what can be observed in everyday life—that conscious contents, including high-level, sophisticated contents, often “just happen”. In the task, the generation of high-level conscious contents (e.g., subvocalizations) are generated involuntarily. This conclusion is consistent with passive frame theory [10]. In the theory, the mechanisms generating conscious contents are themselves unconscious, and so are the mechanisms responding to the contents (which are mechanisms that are distinct from the systems that generate conscious contents). In short, in a form of “unidirectional communication”, conscious contents (e.g., a red apple and an urge) are “sampled” only by action systems, which are themselves unconscious. In the theoretical framework, one conscious content does not, in a sense, “know” of (a) the nature of other conscious contents in the conscious field nor of (b) the nature of ongoing behavior and whether or not the content is relevant to ongoing behavior. It has been proposed that this form of “built-in ignorance” on the part of the cognitive apparatus is actually adaptive (see [88],[89]).

### 4.3. Limitations of the present approach

In these kinds of experiments about the occurrence of conscious thought, it is difficult to avoid the technique of self-report. As mentioned above, this technique has well-known limitations. For example, self-reports can be inaccurate as a result of subjects basing their reports on a strategy of how to comport oneself during an experiment (see discussion in Morsella et al. [34]). In addition, inaccurate memories of fleeting conscious contents can lead to incorrect self-reports [33]. Given the robustness and reliability of the RIT phenomenon and the aforementioned data from the RIT including the rhyming task [40], we do not believe that these well known limitations undermine the validity of the present findings.

### 4.4. Concluding comments

While keeping the shortcomings of the RIT in mind, it is important to reiterate that the RIT is the kind of paradigm that, because it builds incrementally on a robust phenomenon, has of recent been encouraged by leading researchers in the field (e.g., [74],[75]).

The component processes of the RIT are of interest in disparate subfields of the study of mind and brain, including consciousness, attention, decision making, cognitive control, imagery, psychopathology and action control. Because much is known about the neural correlates of many of the component processes underlying the RIT effect, the paradigm is a rich and fecund experimental approach for hypothesis-testing investigations in the field of neuroscience. The paradigm is also perfectly suited for scanner-based neuroimaging research, because it involves a simple procedure for presenting stimuli, and because the dependent measure does not require complicated movements on the part of the subject. More generally, the RIT reveals that the generation of conscious contents, one of the greatest mysteries in science [3],[76],[77], can be studied experimentally.

## Supplementary table

**Table 1.** List of visual objects (line drawings).

Ambulance	Knife
Angel	Lightning
Axe	Lion
Ball	Lips
Balloon	Mosquito
Bed	Motorcycle
Bicycle	Necklace
Bird	Noose
Bomb	Paintbrush
Bullet	Pumpkin
Butterfly	Rabbit
Cake	Rainbow
Candy	Ring
Cannon	Snowflake
Cigarette	Snowman
Claws	Star
Cockroach	Sun
Coffin	Swan
Crown	Swing
Devil	Top
Dog	Tree
Dynamite	Trophy
Fire	Wagon
Fireworks	Waterfall
Flower	World
Fly	Poison
Gravestones	Razor
Grenade	Robber
Guillotine	Scorpion
Guitar	Shark
Gun	Snake
Heart	Spider
House	Tank
Jail	Thorn
Jaws	Tiger
Jewel	Tornado
Kite	Volcano
Kitten	Wasp

## Footnotes

1 A “conscious content” is anything that one is aware of [5]; for example, it might be a color, an urge or a spontaneous autobiographical memory. The “conscious field” is all that one is aware of at one moment in time, which is the combination of all activated conscious contents [6]–[8].

2 Theorists have posited that conscious contents arise involuntarily because of the “encapsulated” nature of the generation of most conscious contents [10],[19]. This encapsulation is evident in perception and also in the generation of action-related urges. In certain stimulus environments, these urges (e.g., to inhale while holding one's breath while underwater) are triggered in a predictable and insuppressible manner [20]. The urges cannot be modulated or turned off voluntarily, even when doing so would be adaptive [20],[21]. The action-related urges are externally-triggered and encapsulated from volitional processes. As noted by Bargh and Morsella [22], these action-related inclinations can be *behaviorally suppressed*, but they often cannot be *mentally suppressed*.

3 In another complex version of the task [29], subjects were presented with a single, focal object and instructed to (a) not subvocalize the name of the visual object; (b) not subvocalize the number of letters in the object name. On a considerable proportion of the trials (0.30 [*SE* = 0.04]), subjects reported experiencing both kinds of imagery. Importantly, the occurrence of both involuntary thoughts reflects the involvement of two very different kinds of unintentional cognitive processes: *object naming* versus *letter counting*. Each of these processes is quite sophisticated and high-level, yielding outputs (e.g., the phonological forms “sun” and “three”) that are not direct reflections of external stimuli.

4 Ironic effects arise when one is more likely to think about a given thing when attempting to not think about that thing. Wegner [41] proposes that these effects arise from an interaction between two distinct processes. One process is an *operating* process, which is associated with the conscious intention to maintain a particular mental state. This process actively scans mental contents (e.g., thoughts, sensations) that can help maintain the desired mental state (e.g., to be calm). This process tends to be effortful, capacity-limited and consciously mediated [41]. The other mechanism is an “ironic” *monitoring* process that automatically scans activated mental contents to detect contents signaling the failure to establish the desired mental state. When the monitor detects contents that signify failed control of the operating mechanism, it increases the likelihood that the particular content will enter consciousness, so that the operating mechanism can then process the content and change its own operations accordingly. The ironic monitor mechanism is usually unconscious, autonomous and requires little mental effort. Harmony between the two kinds of processes fails when the goal in mental control is to *not* activate a particular mental content (e.g., content *X*), because (a) the operating process can bring only goal-related contents into consciousness and cannot actively exclude contents; (b) the ironic monitor will reflexively bring into consciousness mental contents (e.g., content *X*) that are incongruent with the goal. Hence, there will be the automatic activation of content *X* in consciousness (for reviews of ironic processing and thought suppression, see [16],[46]). One difference between the involuntary subvocalization that constitutes the RIT effect and the kinds of effects that have been obtained in most experiments concerning ironic processing is that, in the latter, subjects are presented with a verbal description (e.g., verbal instructions such as “*Do not think of white bears*”), and then the subjects experience involuntary perceptuo-semantic imagery. In the RIT, however, the stimuli are visual and it is the involuntary imagery that is phonological in nature. One could state that the RIT involves the opposite direction of activation of that found in the classic studies on ironic processing.

5 Two other findings complement the Incidental Naming condition. First, subvocalizations toward the visual objects in an RIT [44] arose even when subjects, before being presented with a visual object, performed a block of trials of a task involving another, very different kind of action set (e.g., the Stroop task [17]). Second, the kind of involuntary entry into consciousness found in the RIT arises in tasks that lack any kind of negative instruction to not perform some kind of mental operation. For example, involuntary entry of contents into consciousness arises for ambiguous objects (e.g., Necker cube). In one experiment [45], subjects were instructed to hold in mind, for as long as possible, one way of perceiving an ambiguous object (e.g., Necker cube). Importantly, subjects were never told to *not* think about alternative ways in which the object could be perceived. Involuntary “perceptual reversals” involving involuntary entry into consciousness of the rivalrous percept for a given object, occurred on around 80% of the trials, with roughly three such reversals per 30-second trial.

6 Because prior RIT research [73] suggests that the valence of a stimulus (that is, positive versus negative valence) might, under some circumstances, influence the nature of the RIT effect, and because our stimuli stem in large part from the stimulus set used by Pugh et al. [73], we took the opportunity to have an equal number of objects that fall within the continua of positive valence and negative valence, with each stimulus array having an equal number of objects from each continuum. Because (a) the valence-related data have no bearing on the question here under investigation; (b) the influence of valence on RIT effects is far from straightforward (see discussion in Cho [23]), and (c) at least at this stage of understanding, such valence effects are not worthy of report, we will not discuss the matter of stimulus valence any further. For additional information about the nature of the valence of the stimuli and of potential valence effects, see [73].

7 At the conclusion of the experimental session, subjects completed a series of psychological questionnaires (e.g., Generalized Anxiety Disorder 7-item scale [81]) to assess if inter-individual differences such as high levels of anxiety and depression interact with valence of the stimuli (see discussions of these data in Cho [82]). For present purposes, these data are unrelated to the focus of the current project and have no bearing on the data here under investigation. Hence, they will not be discussed any further. For further information regarding inter-individual measures and the valence of the stimuli, see Cho [82].
